# An Intervention to Improve Cause-of-Death Reporting in New York City Hospitals, 2009–2010

**DOI:** 10.5888/pcd9.120071

**Published:** 2012-10-18

**Authors:** Ann Madsen, Sayone Thihalolipavan, Gil Maduro, Regina Zimmerman, Ram Koppaka, Wenhui Li, Victoria Foster, Elizabeth Begier

**Affiliations:** Author Affiliations: Sayone Thihalolipavan, Gil Maduro, Regina Zimmerman, Wenhui Li, Victoria Foster, Elizabeth Begier, New York City Department of Health and Mental Hygiene, New York, New York; Ram Koppaka, New York City Department of Health and Mental Hygiene, New York, New York, and Centers for Disease Control and Prevention, Atlanta, Georgia.

## Abstract

**Introduction:**

Poor-quality cause-of-death reporting reduces reliability of mortality statistics used to direct public health efforts. Overreporting of heart disease has been documented in New York City (NYC) and nationwide. Our objective was to evaluate the immediate and longer-term effects of a cause-of-death (COD) educational program that NYC’s health department conducted at 8 hospitals on heart disease reporting and on average conditions per certificate, which are indicators of the quality of COD reporting.

**Methods:**

From June 2009 through January 2010, we intervened at 8 hospitals that overreported heart disease deaths in 2008. We shared hospital-specific data on COD reporting, held conference calls with key hospital staff, and conducted in-service training. For deaths reported from January 2009 through June 2011, we compared the proportion of heart disease deaths and average number of conditions per death certificate before and after the intervention at both intervention and nonintervention hospitals.

**Results:**

At intervention hospitals, the proportion of death certificates that reported heart disease as the cause of death decreased from 68.8% preintervention to 32.4% postintervention (*P* < .001). Individual hospital proportions ranged from 58.9% to 79.5% preintervention and 25.9% to 45.0% postintervention. At intervention hospitals the average number of conditions per death certificate increased from 2.4 conditions preintervention to 3.4 conditions postintervention (*P* < .001) and remained at 3.4 conditions a year later. At nonintervention hospitals, these measures remained relatively consistent across the intervention and postintervention period.

**Conclusion:**

This NYC health department’s hospital-level intervention led to durable changes in COD reporting.

## MEDSCAPE CME

Medscape, LLC is pleased to provide online continuing medical education (CME) for this journal article, allowing clinicians the opportunity to earn CME credit.

This activity has been planned and implemented in accordance with the Essential Areas and policies of the Accreditation Council for Continuing Medical Education through the joint sponsorship of Medscape, LLC and Preventing Chronic Disease. Medscape, LLC is accredited by the ACCME to provide continuing medical education for physicians. 

Medscape, LLC designates this Journal-based CME activity for a maximum of 1 **AMA PRA Category 1 Credit(s)™**. Physicians should claim only the credit commensurate with the extent of their participation in the activity.

All other clinicians completing this activity will be issued a certificate of participation. To participate in this journal CME activity: (1) review the learning objectives and author disclosures; (2) study the education content; (3) take the post-test with a 70% minimum passing score and complete the evaluation at www.medscape.org/journal/pcd (4) view/print certificate.

**Release date: October 17, 2012; Expiration date: October 17, 2013**

### Learning Objectives

Upon completion of this activity, participants will be able to:

- Distinguish common mistakes made in completing a death certificate
- Identify appropriate causes of death
- Analyze an intervention to improve the accuracy of a death certificate

**CME EDITOR**

Rosemarie Perrin, Editor; Caran Wilbanks, Editor, *Preventing Chronic Disease*. Disclosure: Rosemarie Perrin and Caran Wilbanks have disclosed no relevant financial relationships.

**CME AUTHOR**

Charles P. Vega, MD, Health Sciences Clinical Professor; Residency Director, Department of Family Medicine, University of California, Irvine. Disclosure: Charles P. Vega, MD, has disclosed no relevant financial relationships.

**AUTHORS AND CREDENTIALS**

Disclosures: Ann Madsen, PhD, MPH; Sayone Thihalolipavan, MD, MPH; Gil Maduro, PhD; Regina Zimmerman, PhD; Ram Koppaka, MD, PhD; Wenhui Li, PhD; Victoria Foster, MPH; Elizabeth Begier, MD, MPH have disclosed no relevant financial relationships.

Affiliations: Ann Madsen, Sayone Thihalolipavan, Gil Maduro, Regina Zimmerman, Ram Koppaka, Wenhui Li, Victoria Foster, Elizabeth Begier, NYC Department of Health & Mental Hygiene, New York, NY.

## Introduction

Inaccurate reporting of cause of death (COD) on death certificates limits the validity and usefulness of mortality indicators for policy, research, and applied public health decisions (1,2). Validation studies and audits have found that heart disease is overreported as a COD (3–7). A comparison of certificates of in-hospital deaths with medical charts for deaths in 2003 in New York City (NYC) showed 91% overreporting of heart disease. Overreporting increased with age by 51% for decedents aged 35 to 74 years, 94% for those aged 75 to 84 years, and 137% for those 85 years or older (7). A previous study of 4 other regions found that 20% of certificates of in-hospital death incorrectly documented heart disease as the underlying COD (6). Because NYC’s heart disease risk factors are not greater than those of the rest of nation (8), overreporting likely partially explains NYC’s high heart disease death rates (9,10).

Physicians typically report COD for deaths from natural causes. Overreporting may result from lack of training, hospital leadership’s failure to emphasize the importance of correctly reporting COD, and survivors’ desire to avoid certain diagnoses (11–15). Physicians are not usually trained in COD reporting despite existing training materials and recommendations that such training be conducted (16–19). Autopsy, physician review panels, and querying (ie, contacting certifiers for clarification) may improve reporting but may not be feasible or cost-effective and therefore may not be adopted (11,20,21). Previous interventions consisting of workshops and interactive training conducted by clinical colleagues have demonstrated short-term improved COD reporting accuracy among trainees (13,14,22–26). Previous reports have not assessed long-term changes in COD reporting or the effect of such changes on population mortality statistics. Our objective was to evaluate immediate and longer-term effects of an educational program in COD documentation that the NYC Department of Health and Mental Hygiene (DOHMH) conducted at 8 NYC hospitals; the evaluation focused on heart disease reporting and the average conditions per death certificate, both of which are indicators of quality of COD reporting.

## Methods

We used a time series design to evaluate an intervention to educate physicians on COD reporting that we conducted from June 2009 to January 2010 at 8 NYC hospitals. The intervention and analysis did not pose any risk to living subjects and was conducted as a quality improvement activity; therefore, it did not require institutional review board approval under the NYC Health Code.

### Identification of intervention hospitals

In early 2009, DOHMH ranked the 64 NYC hospitals reporting more than 50 deaths per year by their 2008 proportion of heart disease deaths; that is, the ratio of heart disease deaths to total deaths that the hospital reported. We selected 8 hospitals that had the greatest potential to improve citywide vital statistics: 7 hospitals with the highest proportions of heart disease deaths and the hospital with the tenth highest proportion because it was the third largest hospital in NYC and had nearly 1,000 deaths per year. All 8 hospitals agreed to participate. The proportion of death certificates reporting heart disease as COD at these 8 hospitals ranged from 60.4% to 78.2% in 2008, while the average at nonintervention hospitals was 25.2% (Table 1). We compared demographics at intervention and nonintervention hospitals in 2008 using *z* scores.

**Table 1:** Selected Characteristics of Intervention and Nonintervention Hospitals in 2008 in an Intervention to Improve Cause-of-Death Reporting in New York City Hospitals, 2009–2010

Hospital	Deaths Per Year	% HD Deaths	Average No. of Conditions^a^	% Non-Hispanic White	Average Age of Decedent, y
Hospital 1	801	78	2.3	78	75
Hospital 2	366	72	2.2	40	76
Hospital 3	540	71	2.4	73	79
Hospital 4	350	70	2.5	80	81
Hospital 5	445	67	2.4	61	74
Hospital 6	601	67	2.7	57	77
Hospital 7	297	66	1.7	91	76
Hospital 8	1,197	60	2.5	79	75
All intervention hospitals	4,597	68	2.4	71	76
All nonintervention hospitals	30,736	25	3.0	43	69
*P* value^b^	NA	<.01^c^	<.01	<.01^c^	<.01

Abbreviations: HD, heart disease; NA, not applicable.

^a^ Based on entity axis codes, ICD-10 codes assigned to the conditions the physician wrote on the death certificate, processed according to the National Center for Health Statistics’ Mortality Medical Data System (MMDS) software algorithm (32).

^b^ *P* values calculated using 2-tailed test. All tests of difference use a standard normal approximation of the sampling distribution of the estimates with their associated *z* scores.

^c^ Pooled estimate of the hypothesized true proportion in the null hypothesis of no difference.

### Underlying COD

The underlying COD, as the World Health Organization defines it, is “the disease or injury that initiated the chain of events leading directly to the death, or the circumstances of the accident or violence which produced the fatal injury” (27). An International Classification of Diseases (ICD)-10 code is assigned as the underlying COD for each death certificate. ICD-10 codes are determined by applying the standardized 1992 World Health Organization *International Classification of Diseases, 10th Revision* (ICD-10) algorithm to the cause-of-death text provided by certifying physicians in COD fields (27). The algorithm is applied automatically by the National Center for Health Statistics’ Mortality Medical Data System (MMDS) software (National Center for Health Statistics, Hyattsville, Maryland) or manually by a trained nosologist if automated coding fails or is not available (28). We defined heart disease deaths as those assigned ICD-10 codes I00–I09, I11, I13, or I20–I51 (29), which is consistent with National Center for Health Statistics’ criteria. These include heart failure, cardiomyopathy, arrhythmias, and acute and chronic rheumatic, hypertensive, ischemic, and pulmonary heart disease.

### Intervention

The intervention consisted of 2 main components: a conference call with senior hospital staff, which included medical directors and medical, quality assurance, admitting, and regulatory affairs staff, and an on-site, in-service training of hospital and clerical staff involved in death certification. Other activities included process mapping of death certification and registration workflow, auditing medical records, and promoting an online learning module. A conference call and in-service training were held for each hospital. The first conference call at any hospital was held on June 26, 2009; the last in-service training was conducted January 13, 2010.

During each conference call, DOHMH described unexpected differences between frequencies of key causes of death (eg, heart disease, Alzheimer disease) reported at the hospital compared with frequencies NYC-wide and nationally. DOHMH stressed the importance of accurate COD reporting for policy and research and the legal requirement for data accuracy in NYC’s Health Code. DOHMH outlined steps required to address the problem. Hospital personnel agreed to complete the remaining intervention activities.

For process mapping, each hospital documented their death registration workflow following the conference call. DOHMH then proposed hospital-specific action plans, including the identification of staff required to attend in-service training and the revision of hospital policy and protocols.

To highlight deficiencies in COD documentation at each hospital, DOHMH identified a sample of 30 death certificates that the hospital registered preintervention. The sample consisted of 10 randomly sampled certificates in each of 3 categories: certificates reporting only a single heart disease condition as the COD, certificates reporting a heart disease condition and other comorbidities as COD, and certificates that did not include heart disease as a cause of death. We asked hospital staff trained in COD documentation to review corresponding medical charts and report all conditions indicated in the medical record as contributing to the death. We compared medical record information with the death certificate and cited the discrepancies we discovered in the in-service training. We also used audit information to inform the average-number-of-conditions outcome measure as described below.

DOHMH requested that physicians and staff involved in death certification at intervention hospitals complete the Improving Cause of Death Reporting online training module created by DOHMH (30).

DOHMH quality improvement and medical personnel conducted an in-service training with physicians and staff involved in death registration at each intervention hospital. The 45-minute presentation, typically attended by 30 to 100 residents and staff, addressed legal requirements for death registration; compared COD distributions at the hospital, throughout NYC, and nationwide; gave examples of discrepancies between death certificate cause of death and the medical record identified during each hospital’s audit; and provided generic examples of proper and improper COD documentation, with emphasis on heart disease. A question-and-answer session followed, and we distributed DOHMH’S *City Health Information* (31) bulletin on improving cause of death reporting.

We defined 2 primary outcome measures: 1) the proportion of heart disease deaths reported on death certificates, which is an indicator of the intervention’s effect on heart disease overreporting, and 2) the average number of conditions reported on death certificates, defined as the number of entity axis codes documented in Parts I and II of the certificate. Entity axis codes are ICD-10 codes assigned to the conditions the physician wrote on the death certificate, processed according to the MMDS algorithm. The algorithm processes and codes the conditions reported by the physician in the cause of death section while eliminating redundancies and inconsistencies (32). On the basis of the DOHMH audit, the number of entity axis codes is an indicator of sufficient detail and COD reporting quality. We classified audited death certificates as inaccurate if the underlying COD on the death certificate was not reported in the medical record or as accurate if the underlying COD on the death certificate was reported in the medical record. Inaccurate death certificates on average reported fewer conditions compared with accurate certificates (1.45 vs 1.75, *P* = .07, n = 147, *t* test 2-sample equal variance).

For statistical analysis we defined the preintervention period as January 1, 2009, through June 30, 2009; the active intervention period as June 30, 2009, through December 31, 2009; the postintervention period as January 1, 2010, through June 30, 2010; and the extended postintervention period as January 1, 2011, through June 30, 2011. We used January–through–June deaths each year to control for seasonal variation in COD. The primary analysis evaluated change in the 2 outcome measures at intervention hospitals between the pre- and postintervention periods and the preintervention and extended postintervention periods by using a *z* score for the difference between 2 proportions with pooled variance estimates. Secondary analyses repeated these outcome measure comparisons within nonintervention hospitals and citywide. To understand any trends unrelated to the intervention, we calculated the outcome measures for each calendar year between 2001 and 2010 at intervention hospitals individually and combined, at nonintervention hospitals, and citywide. We used SAS version 9.2 (SAS Institute, Inc, Cary, North Carolina) for all analyses.

## Results

In 2008, the 8 intervention hospitals reported 28% of heart disease deaths and 13% of all-cause deaths among all NYC hospitals with more than 50 deaths per year. At intervention hospitals, the number of deaths ranged from 297 to 1,197 for a total of 4,597; the proportion of heart disease deaths ranged from 60.4% to 78.1%, averaging 68.2%; and the average number of conditions reported per death certificate ranged from 1.7 to 2.7, averaging 2.4 (Table 1). Nonintervention hospitals reported 30,736 deaths in 2008 with 25.2% due to heart disease and reported an average of 3.0 conditions per certificate. Decedents at nonintervention hospitals were, on average, younger and less likely to be non-Hispanic white than at intervention hospitals.

At intervention hospitals the proportion of heart disease deaths decreased from 68.8% preintervention to 32.4% postintervention (*P* < .01). At each hospital, the proportion ranged from 58.6% to 79.5% in the preintervention period and from 25.9% to 45.0% in the postintervention period. The decrease was significant (*P* < .01) at each hospital; the absolute difference ranged from 27.8 to 52.4 percentage points (Table 2). The average proportion of heart disease deaths in the extended postintervention period ranged from 23.5% to 50.0%, significantly lower than preintervention at each hospital. In comparison, the proportion of heart disease deaths at nonintervention hospitals remained relatively consistent: 26.6% preintervention, 24.4% postintervention, and 22.1% extended postintervention. Citywide, the proportion of heart disease deaths decreased from 39.1% preintervention to 34.2% postintervention and to 32.5% for the extended postintervention period.

**Table 2:** Percentage of Death Certificates Reporting Heart Disease as Cause of Death at Intervention and Nonintervention Hospitals, New York City, 2009–2011

Variable	Jan–Jun 2009, %	Jan–Jun 2010, %	Percentage Point Change 2009–2010	*P* Value^a^	Jan-Jun 2011, %	Percentage Point Change 2009–2011	*P* Value^a^
Intervention hospitals	68.8	32.4	−36.4	<.001	33.7	−35.1	<.001
Hospital 1	79.5	27.0	−52.4	<.001	23.5	−55.9	<.001
Hospital 2	70.5	34.1	−36.4	<.001	35.9	−34.6	<.001
Hospital 3	72.6	42.5	−30.1	<.001	44.3	−28.3	<.001
Hospital 4	72.8	45.0	−27.8	<.001	50.0	−22.8	<.001
Hospital 5	58.6	26.2	−32.4	<.001	26.1	−32.5	<.001
Hospital 6	68.2	36.9	−31.2	<.001	39.1	−29.1	<.001
Hospital 7	74.9	35.2	−39.8	<.001	33.8	−41.2	<.001
Hospital 8	58.9	25.9	−32.9	<.001	39.5	−19.4	.001
Nonintervention hospitals	26.6	24.4	−2.2	<.001	22.1	−4.5	<.001
**Citywide**	39.1	34.3	−4.8	<.001	32.5	−6.6	<.001

^a^ *P* values calculated by using a 2-tailed test. All tests of difference use a standard normal approximation of the sampling distribution of the estimates with their associated *z* scores to test the null hypothesis of equal proportions with common variance estimated by pooled proportion.

At intervention hospitals combined, the average number of conditions reported increased from 2.4 preintervention to 3.4 postintervention (Table 3). The absolute increase at each hospital in the preintervention period ranged from 0.75 to 1.33 conditions on average. The average number of conditions reported in the extended postintervention period remained higher at each intervention hospital, an average of 3.4 across hospitals. In comparison, nonintervention hospitals reported a relatively consistent average number of conditions: 2.9 preintervention, 2.9 postintervention, and 3.0 extended postintervention. Citywide, the average number of conditions reported per death certificate increased from 2.7 preintervention to 2.8 postintervention and 2.9 extended postintervention.

**Table 3:** Average Number of Conditions Reported per Death Certificate at Intervention and Nonintervention Hospitals, New York City, 2009–2011

Variable	Jan–Jun 2009	Jan–Jun 2010	Change 2009–2010	*P* Value^a^	Jan-Jun 2011	Change 2009-2011	*P* Value^a^
Intervention hospitals	2.35	3.39	1.04	<.001	3.38	1.03	<.001
Hospital 1	2.28	3.33	1.05	<.001	3.48	1.20	<.001
Hospital 2	1.87	2.95	1.08	<.001	3.17	1.30	<.001
Hospital 3	2.41	3.74	1.33	<.001	3.66	1.25	<.001
Hospital 4	2.58	3.53	0.95	<.001	3.88	1.30	<.001
Hospital 5	2.52	3.39	0.87	<.001	2.91	0.39	<.001
Hospital 6	2.42	3.16	0.74	<.001	3.41	0.99	<.001
Hospital 7	2.50	3.67	1.17	<.001	4.31	1.81	<.001
Hospital 8	1.52	2.76	1.24	<.001	2.16	0.64	<.001
Nonintervention hospitals	2.89	2.88	−0.01	.441	3.00	0.11	<.001
**Citywide**	2.74	2.84	0.10	<.001	2.93	0.19	<.001

^a^ *P* values calculated by using a 2-tailed test. All tests of difference use a standard normal approximation of the sampling distribution of the estimates with their associated *z* scores to test the null hypothesis of equal means.

**Figure 1:**
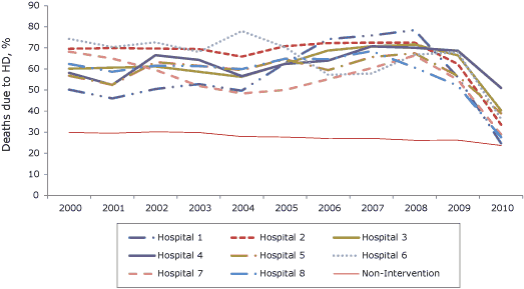
Percentage of deaths attributed to heart disease at each of the 8 intervention hospitals and at all nonintervention hospitals combined, New York City, 2000–2010.

**Figure 2:**
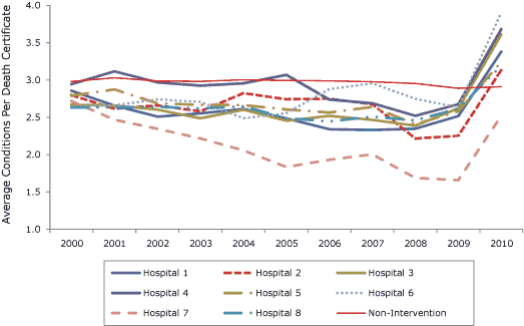
Average number of conditions reported per death at each of the 8 intervention hospitals and at all nonintervention hospitals combined, New York City, 2000–2010.

The proportions of deaths from heart disease had been stable for several years before this intervention at both intervention hospitals and nonintervention hospitals (Figure 1). Similarly, the magnitude of change in the average number of conditions reported was unprecedented at any of the hospitals before the intervention and similar among intervention hospitals following the intervention (Figure 2).

**Table:** 

Year	Hospital 1	Hospital 2	Hospital 3	Hospital 4	Hospital 5	Hospital 6	Hospital 7	Hospital 8	Nonintervention
**2000**	50.176	69.512	60.075	58.108	56.745	74.260	68.161	62.324	29.878
**2001**	46.026	69.916	60.606	52.368	52.419	70.342	64.907	58.621	29.541
**2002**	50.445	69.613	61.155	66.497	63.269	72.583	59.524	61.532	30.208
**2003**	52.806	69.388	58.639	64.269	61.495	68.012	51.908	61.325	29.835
**2004**	49.672	65.819	56.178	56.522	59.420	77.922	48.375	59.887	27.932
**2005**	62.694	70.755	62.395	62.353	64.542	70.255	50.000	64.761	27.726
**2006**	74.142	72.222	68.715	63.950	59.368	57.165	55.108	64.645	26.997
**2007**	75.809	72.384	70.723	70.621	65.596	57.790	60.357	68.416	27.099
**2008**	78.402	72.404	71.481	70.000	67.416	66.722	66.330	60.652	26.152
**2009**	56.046	62.278	66.381	68.627	56.592	67.862	54.861	52.007	26.199
**2010**	24.756	33.579	40.285	50.949	38.914	36.260	28.814	27.606	23.714

**Table:** 

Year	Hospital 1	Hospital 2	Hospital 3	Hospital 4	Hospital 5	Hospital 6	Hospital 7	Hospital 8	Nonintervention
**2000**	2.86	2.80	2.67	2.94	2.79	2.64	2.72	2.63	2.98
**2001**	2.66	2.62	2.66	3.12	2.88	2.66	2.47	2.63	3.03
**2002**	2.51	2.66	2.60	2.97	2.69	2.74	2.35	2.64	2.99
**2003**	2.55	2.58	2.49	2.93	2.67	2.71	2.22	2.62	2.98
**2004**	2.61	2.82	2.60	2.96	2.66	2.49	2.06	2.65	3.00
**2005**	2.49	2.74	2.45	3.07	2.61	2.56	1.83	2.49	3.00
**2006**	2.34	2.75	2.52	2.74	2.56	2.88	1.93	2.45	2.99
**2007**	2.33	2.68	2.47	2.69	2.64	2.96	2.00	2.51	2.98
**2008**	2.35	2.22	2.39	2.52	2.41	2.75	1.69	2.46	2.95
**2009**	2.52	2.25	2.62	2.68	2.58	2.63	1.66	2.62	2.89
**2010**	3.38	3.14	3.61	3.68	3.24	3.90	2.52	3.19	2.91

## Discussion

This hospital-level intervention is the first to demonstrate immediate and durable changes in COD reporting with a reduction in heart disease overreporting and an increase in the average number of conditions reported. The reporting changes at the 8 intervention hospitals were so pronounced that citywide outcome measures were notably different before and after the intervention.

Fourteen previous reports have measured effectiveness of COD educational efforts (33); only 1 study was conducted in the United States (21). Some studies asked trainees to complete death certificates for fabricated cases immediately pre- and postintervention, an approach that limits generalizability to other causes of death and does not evaluate sustained intervention effects (33). Other studies compared COD documented on the death certificate with the medical record before and after intervention, a resource-intensive approach. In those reports, hospital-affiliated physicians provided training (33). We add to the literature by showing that public health staff vested in vital statistics data can also effect change and that interactive training can have a sustained effect on COD reporting.

Our study has some limitations. The average number of conditions reported was correlated with data accuracy preintervention, but this may not be generalizable to other settings. Our intervention hospitals had significant heart disease overreporting; thus, our findings may not be generalizable to hospitals with a lesser degree of overreporting or different types of quality issues. Another limitation is that we did not query physicians postintervention to learn whether the in-service training met their COD reporting needs.

Given the volume of deaths occurring at intervention hospitals (375 per month on average), resources did not permit direct comparison of death certificates with the medical record except in our preintervention sample. The inability to definitively conclude postintervention accuracy is a study limitation. We did establish that hospitals overreporting heart disease preintervention reported fewer conditions on average and that inaccurate death certificates of overreporting hospitals reported fewer conditions than their accurate death certificates reported. Thus, the observed increase in average number of conditions reported at intervention hospitals suggests improved COD reporting. Additionally, the number of deaths from other leading causes postintervention increased proportionately as expected, suggesting improved reporting rather than a shift from heart disease to a single or few random erroneous reported causes of death (34). An alternate explanation is that deaths inaccurately reported preintervention as heart disease continued to be reported inaccurately, but in proportion to NYC’s leading causes of death, which seems less likely.

Our analyses of 10-year trends in these outcome measures and our comparison of outcome measures pre- and postintervention at nonintervention hospitals establishes that the decrease in heart disease deaths and the increase in the average number of conditions postintervention is restricted to intervention hospitals and is not likely due to a secular or historical trend. Although the decrease in heart disease proportions was statistically significant in the nonintervention group, because approximately 14,000 deaths occurred during each observation period, the decrease is an order of magnitude less than at intervention hospitals. The slight decrease in heart disease deaths and increase in average number of conditions reported at nonintervention hospitals between postintervention and extended postintervention periods may reflect DOHMH’s continued efforts to improve COD reporting citywide.

Although bias or confounding might explain results in any nonrandomized study, neither can fully explain our results, unless the true heart disease death rates decreased to this degree in populations served by intervention hospitals only during our study period, and we coincidentally embarked on a campaign to improve COD reporting during this period. Another possible but unlikely explanation is regression to the mean; that is, because we selected hospitals based on their high percentages of deaths from heart disease, by chance this percentage was closer to the average upon second measurement. Previous years’ data do not support this explanation because intervention hospitals historically had reported high proportions of heart disease and nonintervention hospitals had reported low proportions.

Decedents at intervention hospitals were older and more likely to be non-Hispanic white than at nonintervention hospitals. While heart disease risk also varies by these factors and COD reporting quality varies by age, this variation cannot explain the intervention’s positive results. We compared the recent outcome measures over time within each intervention hospital so that differences in decedents’ characteristics by hospital are not confounding our primary comparison. Furthermore, an analysis of changes in all reported causes of death following the intervention and controlling for decedents’ characteristics did not alter the intervention’s observed effect (34).

This intervention incorporated hospital-specific policy, practices, and educational components to achieve the support of staff and administration and was completed at little cost. The primary expenditure, outside of the staff time of DOHMH personnel, consisted of minimal travel expenses. One full-time epidemiologist devoted approximately 50% of her time to developing content over the course of 18 months with input from subject matter experts. The director of the Office of Vital Statistics conducted the conference calls. The director and a DOHMH physician conducted the in-service trainings. Health departments with fewer resources or those that cover a larger geographic area may be able to improve COD reporting quality without extensive travel by using a conference call alone.

One key benefit of on-site, in-service training was qualitative feedback from a larger audience. Most doctors reported no prior training in death certification. Additionally, physicians and staff expressed frustration over the past DOHMH practice of rejecting certificates based on COD and suggested that funeral directors, affected by death registration delays, may proactively request certain “safe” causes of death, such as atherosclerotic heart disease, to avoid DOHMH rejections. Physicians also perceived validation checks in the Electronic Death Registration System (EDRS) as obstacles to death registration. DOHMH has reduced these barriers citywide. As of March 2010, the death registration protocol requires rejection only if the cause of death does not appear natural or the only COD reported is a mechanism (eg, cardiopulmonary arrest, asystole, respiratory arrest). As of October 2009, physicians can override many COD related EDRS validation checks. As of January 2010, the NYC Health Code requires all EDRS users to complete an online training on COD documentation, which may explain some postintervention changes in outcome measures among nonintervention and intervention hospitals. However, compliance with this training requirement has been poor, and further efforts are planned to improve enforcement. DOHMH has also discussed the importance of accurate cause of death reporting and physician autonomy at meetings of NYC funeral director associations. These and other citywide efforts may explain some COD reporting changes at intervention hospitals and at nonintervention hospitals.

In NYC, medical residents complete many death certificates. Sustained improvement in COD reporting will depend on hospital and residency administrations’ support of continuous quality improvement. At some hospitals, improvements waned in the expanded postintervention period, indicating that ongoing training is needed to ensure that new staff and medical residents understand COD documentation. On the basis of the success of this intervention, DOHMH conducted conference calls and in-service trainings in 12 additional hospitals in 2011. As resources permit, DOHMH will reach all NYC hospitals. Other completed COD improvement initiatives include issuance of COD data quality reports; dissemination of education materials such as physician pocket cards and COD posters; assisting via telephone during weekdays; and monitoring DOHMH death registration rejections.

National efforts to improve COD reporting quality are ongoing. In partnership with National Association for Public Health Statistics and Information Systems (NAPHSIS) and the National Center for Health Statistics, DOHMH developed an e-learning course on COD completion for national use, which is available to NAPHSIS members. Other jurisdictions can customize the national module.

Inaccurate COD reporting occurs at local, state, and national levels. Many researchers use mortality data; therefore, poor quality COD reporting, including heart disease overreporting, affects the usefulness of public health policies, spending, and programs informed by the data. We have demonstrated that a health department can reduce heart disease overreporting with a training intervention. DOHMH continues to expand COD training in NYC. Other US jurisdictions should consider similar interventions to address this critical national problem.

## Figures and Tables

**Table:** 

**1. The activity supported the learning objectives.**				
**Strongly Disagree**				**Strongly Agree**
1	2	3	4	5
**2. The material was organized clearly for learning to occur.**				
**Strongly Disagree**				**Strongly Agree**
1	2	3	4	5
**3. The content learned from this activity will impact my practice.**				
**Strongly Disagree**				**Strongly Agree**
1	2	3	4	5
**4. The activity was presented objectively and free of commercial bias.**				
**Strongly Disagree**				**Strongly Agree**
1	2	3	4	5

## Post-Test Information

To obtain credit, you should first read the journal article. After reading the article, you should be able to answer the following, related, multiple-choice questions. To complete the questions (with a minimum 70% passing score) and earn continuing medical education (CME) credit, please go to http://www.medscape.org/journal/pcd. Credit cannot be obtained for tests completed on paper, although you may use the worksheet below to keep a record of your answers. You must be a registered user on Medscape.org. If you are not registered on Medscape.org, please click on the "Register" link on the right hand side of the website to register. Only one answer is correct for each question. Once you successfully answer all post-test questions you will be able to view and/or print your certificate. For questions regarding the content of this activity, contact the accredited provider, CME@medscape.net. For technical assistance, contact CME@webmd.net. American Medical Association's Physician's Recognition Award (AMA PRA) credits are accepted in the US as evidence of participation in CME activities. For further information on this award, please refer to http://www.ama-assn.org/ama/pub/category/2922.html. The AMA has determined that physicians not licensed in the US who participate in this CME activity are eligible for **AMA PRA Category 1 Credits™**. Through agreements that the AMA has made with agencies in some countries, AMA PRA credit may be acceptable as evidence of participation in CME activities. If you are not licensed in the US, please complete the questions online, print the AMA PRA CME credit certificate and present it to your national medical association for review.

## Post-Test Questions

### Article Title: An Intervention to Improve Cause-of-Death Reporting in New York City Hospitals, 2009–2010

**CME Questions**

1. You are asked to fill out a death report on an 85-year-old continuity patient who just passed away. As you perform this task, what should you keep in mind as the most commonly overreported condition on death certificates?
  - Heart disease
  - Cancer
  - Lung disease
  - Unknown
2. Which of the following options is most appropriate as a cause of death?
  - Cardiac arrest
  - Respiratory arrest
  - Chronic hypoxia
  - Myocardial infarction
3. You remember the current study designed to improve the accuracy of death reports. What was one of the main interventions in this study?
  - Direct communication between nurses and coroners’ offices on each form
  - One expert individual completing death certificates for multiple hospitals
  - On-site training of hospital and clerical staff on completion of the death certificate
  - Automated completion of the death certificate via the Internet
4. Which of the following statements regarding subgroup analysis of the current study is pertinent for this patient?
  - There was no change in the proportion of heart disease listed on the death certificate or the number of conditions reported
  - There was a decrease in the proportion of heart disease listed on the death certificate and an increase in the number of conditions reported
  - There was an increase in the proportion of heart disease listed on the death certificate only
  - There was a decrease in the number of conditions reported only

**Evaluation**

## References

[1] Kircher T , Anderson RE . Cause of death. Proper completion of the death certificate. JAMA 1987;258(3):349–52. 10.1001/jama.1987.03400030065033 3599328

[2] Messite J , Stellman SD . Accuracy of death certificate completion: the need for formalized physician training. JAMA 1996;275(10):794–6. 10.1001/jama.1996.03530340058030 8598597

[3] Sington JD , Cottrell BJ . Analysis of the sensitivity of death certificates in 440 hospital deaths: a comparison with necropsy findings. J Clin Pathol 2002;55(7):499–502. 10.1136/jcp.55.7.499 12101193 PMC1769693

[4] Kircher T , Nelson J , Burdo H . The autopsy as a measure of accuracy of the death certificate. N Engl J Med 1985;313(20):1263–9. 10.1056/NEJM198511143132005 4058507

[5] Lloyd-Jones DM , Martin DO , Larson MG , Levy D . Accuracy of death certificates for coding coronary heart disease as the cause of death. Ann Intern Med 1998;129(12):1020–6. 9867756 10.7326/0003-4819-129-12-199812150-00005

[6] Coady SA , Sorlie PD , Cooper LS , Folsom AR , Rosamond WD , Conwill DE . Validation of death certificate diagnosis for coronary heart disease: the Atherosclerosis Risk in Communities (ARIC) Study. J Clin Epidemiol 2001;54(1):40–50. 10.1016/S0895-4356(00)00272-9 11165467

[7] Agarwal R , Norton JM , Konty K , Zimmerman R , Glover M , Lekiachvili A , Overreporting of deaths from coronary heart disease in New York City hospitals, 2003. Prev Chronic Dis 2010;7(3):A47. 20394686 PMC2879979

[8] Gwynn RC , Garg RK , Kerker BD , Frieden TR , Thorpe LE . Contributions of a local health examination survey to the surveillance of chronic and infectious diseases in New York City. Am J Public Health 2009;99(1):152–9. 10.2105/AJPH.2007.117010 18556616 PMC2636612

[9] Hoyert DL , Heron MP , Murphy SL , Kung HC . Deaths: final data for 2003. Natl Vital Stat Rep 2006;54(13):1–91. 16689256

[10] New York City Department of Health and Mental Hygiene. Annual mortality data file — 2003. New York (NY): Bureau of Vital Statistics; 2003.

[11] Smith Sehdev AE , Hutchins GM . Problems with proper completion and accuracy of the cause-of-death statement. Arch Intern Med 2001;161(2):277–84. 10.1001/archinte.161.2.277 11176744

[12] Myers KA , Eden D . Death duties: workshop on what family physicians are expected to do when patients die. Can Fam Physician 2007;53(6):1035–8. 17872782 PMC1949219

[13] Myers KA , Farquhar DRE . Improving the accuracy of death certification. CMAJ 1998;158(10):1317–23. 9614825 PMC1229326

[14] Pain CH , Aylin P , Taub NA , Botha JL . Death certification: production and evaluation of a training video. Med Educ 1996;30(6):434–9. 10.1111/j.1365-2923.1996.tb00864.x 9217906

[15] McAllum C , St George I , White G . Death certification and doctors’ dilemmas: a qualitative study of GPs’ perspectives. Br J Gen Pract 2005;55(518):677–83. 16176734 PMC1464060

[16] Writing cause of death statements — basic principles. National Association of Medical Examiners; 2005. http://thename.org/index.php?option=com_content&task=view&id=113&Itemid=58. Accesssed July 20, 2010.

[17] Hanzlick R . Protocol for writing cause-of-death statements for deaths due to natural causes. Arch Intern Med 1996;156(1):25–6. 10.1001/archinte.1996.00440010031005 8526693

[18] Barber JB . Improving accuracy of death certificates. J Natl Med Assoc 1992;84(12):1007–8. 1296990 PMC2571653

[19] National Center for Health Statistics. Physicians’ handbook on medical certification of death. Hyattsville (MD): National Center for Health Statistics; 2004. http://www.cdc.gov/nchs/data/misc/hb_cod.pdf. Accessed September 5, 2012.

[20] Instruction manual part 20: ICD-10 cause-of-death querying, 2007. Hyattsville (MD): National Center for Health Statistics; 2007.

[21] Bangdiwala SI , Cohn R , Hazard C , Davis CE , Prineas RJ . Comparisons of cause of death verification methods and costs in the Lipid Research Clinics Program Mortality Follow-up Study. Control Clin Trials 1989;10(2):167–87. 10.1016/0197-2456(89)90029-9 2666025

[22] Lakkireddy DR , Basarakodu KR , Vacek JL , Kondur AK , Ramachandruni SK , Esterbrooks DJ , Improving death certificate completion: a trial of two training interventions. J Gen Intern Med 2007;22(4):544–8. 10.1007/s11606-006-0071-6 17372807 PMC1839864

[23] Degani AT , Patel RM , Smith BE , Grimsley E . The effect of student training on accuracy of completion of death certificates. Med Educ Online 2009;14:17. 10.3885/meo.2009.Res00315 20165531 PMC2779619

[24] Weeramanthri T , Beresford W , Sathianathan V . An evaluation of an educational intervention to improve death certification practice. Aust Clin Rev 1993;13(4):185–9. 8311787

[25] Pandya H , Bose N , Shah R , Chaudhury N , Phatak A . Educational intervention to improve death certification at a teaching hospital. Natl Med J India 2009;22(6):317–9. 20384023

[26] Selinger CP , Ellis RA , Harrington MG . A good death certificate: improved performance by simple educational measures. Postgrad Med J 2007;83(978):285–6. 10.1136/pgmj.2006.054833 17403959 PMC2600020

[27] Manual of the International Statistical Classification of Diseases, Injuries, and Causes of Death, based on the recommendations of the Tenth Revision Conference, 2010. Geneva (CH): World Health Organization; 2010.

[28] Centers for Disease Control and Prevention. About the Mortality Medical Data System; 2010. Updated January 4, 2010.http://www.cdc.gov/nchs/nvss/mmds/about_mmds.htm. Accesssed December 19, 2010.

[29] International Statistical Classification of Diseases and Related Health Problems 10th Revision [database on the Internet]. World Health Organization; 2007. http://apps.who.int/classifications/apps/icd/icd10online/. Accessed July 23, 2010.

[30] New York City Department of Health and Mental Hygiene. Improving cause of death reporting education module. http://www.nyc.gov/html/doh/media/video/icdr/index.html. Accessed July 6, 2010.

[31] New York City Department of Health and Mental Hygiene. City health information: improving cause of death reporting. 2008;27(9):71–8.

[32] National Bureau of Economic Research. Entity axis codes. Cambridge (MA): National Bureau of Economic Research; 1995. http://www.nber.org/mortality/1995/docs/entity95.txt. Accessed August 28, 2012.

[33] Aung E , Rao C , Walker S . Teaching cause-of-death certification: lessons from international experience. Postgrad Med J 2010;86(1013):143–52. 10.1136/pgmj.2009.089821 20237008

[34] Al-Samarrai T , Madsen A, Zimmerman R, Maduro G, Li W, Greene C, Begier E. Impact of a pilot intervention to decrease overreporting of heart disease death — New York City, 2009-2010. Presented at: 60th Annual Epidemic Intelligence Service (EIS) Conference; April 13, 2011; Atlanta, GA.

